# Application of mediastinal drainage tube in intrathoracic esophageal anastomotic leakage for early diagnosis and effective treatment: a retrospective study

**DOI:** 10.1186/s13019-021-01435-9

**Published:** 2021-03-25

**Authors:** Hainong Ma, Xu Song, Jie Li, Guofang Zhao

**Affiliations:** 1 Hwa Mei Hospital, University of Chinese Academy of Sciences, Ningbo, China; 2 Ningbo Institute of Life and Health Industry, University of Chinese Academy of Sciences, Ningbo, China

**Keywords:** Esophageal cancer, Esophagectomy, Anastomotic leakage

## Abstract

**Background:**

Intrathoracic esophageal anastomotic leakage (AL) is one of the most fatal complications after esophagectomy. In this study, we placed an additional drainage tube in the esophagus bed and evaluated its effect in early diagnosis and treatment of AL.

**Methods:**

From January 2010 to August 2020, 312 patients with esophageal or cardia carcinoma underwent esophageal resection with intrathoracic esophagogastric anastomosis. A total of 138 patients with only one pleural drainage tube were divided into the “Control Group” and 174 patients with a pleural drainage tube and an additional mediastinal drainage tube (MDT) were divided into the “Tube Group”. For all patients, the incidence of postoperative AL, the time to diagnosis, time to recovery, and patient outcome were analyzed.

**Results:**

No significant differences were observed in the AL rate (*P* = 0.837) and postoperative pain between two groups. However, in the Tube Group, almost all the patients were diagnosed prior to the appearance of hyperpyrexia, which was considered as the earliest and most common symptom after AL. In the Tube Group, a significant decrease was observed in the incidence of incurable fistula, which required re-operation or variable treatments under gastroscopy when compared to the Control Group (*P* = 0.032). Finally, patients in the Tube Group showed reduced post AL hospital day (*P* = 0.015) and a lower mortality, however, when compared to the Control Group, no significant differences were observed (*P* = 0.188).

**Conclusions:**

Placement of an MDT does not prevent AL, but it is an effective approach for earlier diagnosis of AL and facilitates fistula healing and patient recovery.

## Background

Esophageal cancer is one of the most serious global health problems, especially in developing countries [1]. Surgery is a major type of treatment for patients with locoregional esophageal cancer [2]. In recent years, improvements in surgical techniques and postoperative care have resulted in a significant reduction in surgical complications, morbidity and mortality [3]. However, as one of the most frequent postoperative complications, anastomotic leakage (AL) is still difficult to completely avoid. According to the literature, AL occurs in 11.4 to 21.2% [4–7] of postoperative patients with esophageal cancer, with an associated mortality rate between 7.2 and 35% [8]. Early diagnosis is critical to facilitate fistula healing and patient recovery, and to decrease the AL-associated mortality [9].

Clinical symptoms of AL, including hyperpyrexia, thoracodynia, chest distress, tachycardia, and increased and feculent liquid from pleural drainage [9–13], are nonspecific and in general appear too late for timely treatment. The main reason is that these symptoms generally emerge secondary to the infection around the fistula, which is already severe and difficult to drain and flush because of unsatisfying drainage. After the onset of symptoms, several diagnostic modalities are available for AL detection, including esophagography, endoscopy, and the observation of methylene blue in the drainage tube after oral administration [9]. This generally occurs a few days later than the occurrence of AL. Therefore, in clinical practice, more effective diagnostic methods for AL are of utmost importance.

In previous studies, in order to effectively treat AL, several treatment methods have been developed, including conservative treatment, endoscopic techniques and a second operation [14, 15]. In general, the principles of these management strategies involve closure of the anastomotic fistula, containment of the leakage, and adequate drainage of fluid collections [9]. Unfortunately, because the abscess cavity resulted from AL is typically very deep, it is a challenge to accurately place a drainage tube without performing a second surgery. Consequently, drainage after AL is often incomplete and the infection around the fistula is difficult to control.

Starting in September 2015, to solve the aforementioned problems, an additional mediastinal drainage tube (MDT) was placed during the operation next to the anastomotic stoma in the esophagus bed. In case of leakage, the MDT would allow for early diagnosis through changes in the drainage fluid, and could completely drain the leakage cavity and remove secretions or necrosis by intermittent flushing. In this study, a ten-year retrospective database was used to analyze early diagnosis and the treatment effect of the MDT.

## Methods

In this study, a ten-year retrospective database was used to identify patients with esophageal or cardia carcinoma who underwent esophageal resection with left or right intrathoracic esophagogastric anastomosis at the Thoracic Surgery Department, Hwa Mei Hospital, University of Chinese Academy of Sciences from January 2010 to August 2020. Exclusion criteria were as follows: (I) patients underwent esophagectomy under Video-Assisted Thoracic Surgery, (II) patients with cervical anastomosis, (III) the initial 10 cases were excluded because of the learning curve effect. In total, there were 312 recorded cases. Among them, 138 patients with only one pleural drainage tube were divided into the “Control Group” and 174 patients with a pleural drainage tube and an additional MDT were divided into the “Tube Group”.

**Fig. 1:**
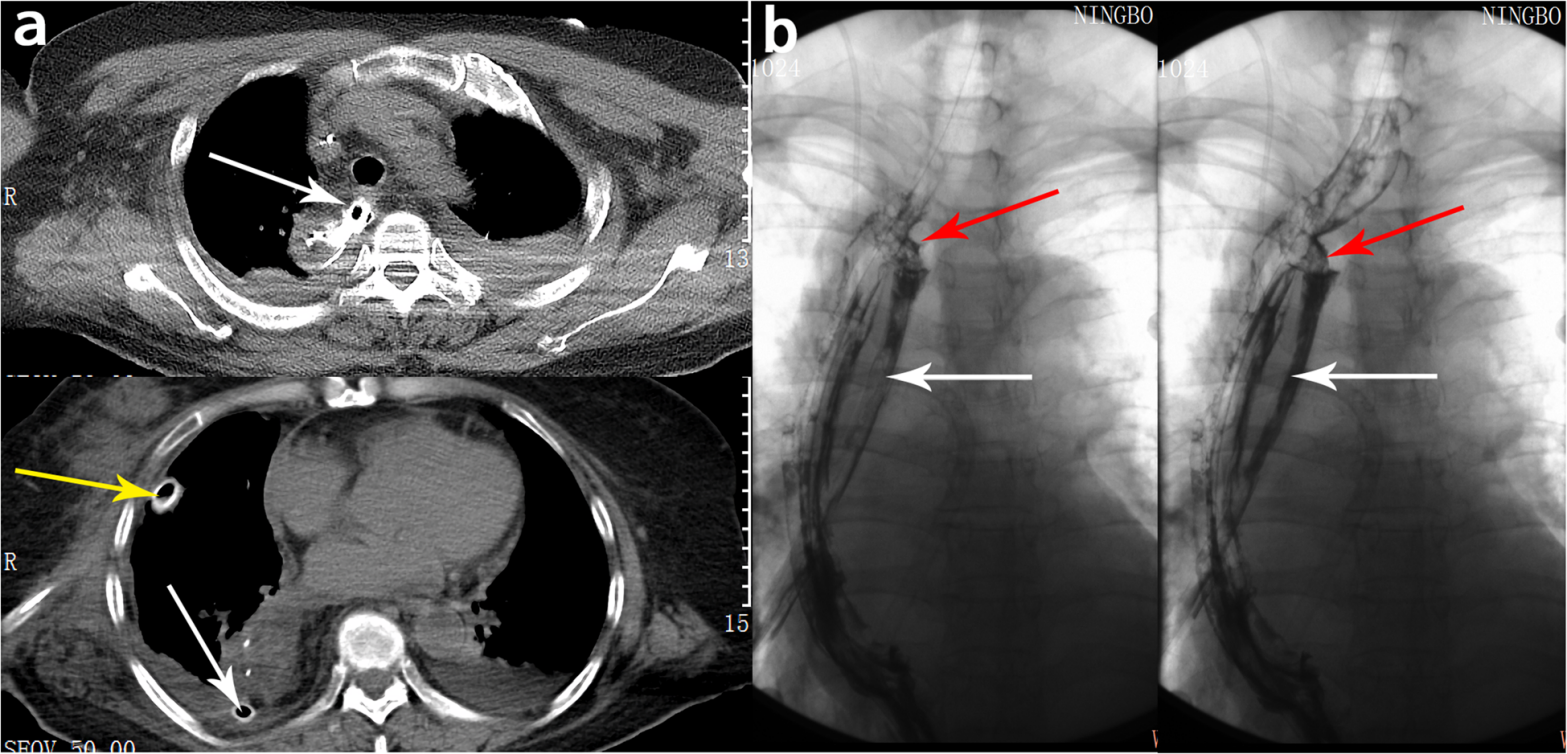
Location of the drainage tubes (**a)** and the drainage effect of the MDT (**b)**. The white arrow indicates the mediastinal drainage tube. The yellow arrow indicates the pleural drainage tube. The red arrow indicates the anastomotic fistula

Preoperative assessments and surgical procedures were performed according to NCCN clinical practice guidelines [3]. After esophagus tumor resection and lymph node dissection, the stomach, which was already made into a narrow tube, was pulled into the pleural cavity for anastomosis using an anastomat. After the diaphragm was sutured, in the Control Group, a 26-Fr drainage tube was placed above the diaphragm into the thoracic cavity that served as a pleural drainage tube. In the Tube Group, in addition to this tube, another 26-Fr drainage tube was placed next to the anastomosis into the esophagus bed as an MDT. The white arrow in Fig. 1a indicates the MDT next to the anastomotic stoma and the yellow arrow indicates the pleural drainage tube above the diaphragm.

Upon return to the ward, the two tubes were connected to water sealed bottles. To assess the position of the tubes, a chest X-ray was performed on postoperative Day 1. In the Control Group, the pleural drainage tube would be retained until the patient was able to eat normally. However, in the Tube Group, the pleural drainage tube was removed when no air leak was observed and the drainage volume of drainage was below 150 ml per day. The mediastinal drainage tube was not removed until the patient was able to eat normally. Esophagography, also known as contrast swallow examination, was performed in patients with suspected AL, and the AL diagnosis was confirmed by both a radiologist and a thoracic surgeon. As shown in Fig. 1b, when the AL occurred, the contrast agent was completely drained out through the MDT, as indicated by the red arrow.

All patients received health education, including a pain score method of a numerical rating scale (NRS) at admission. To assess the postoperative pain score, a chart card with a 10-cm-long horizontal line with word anchors at each end, ranging from 0 = “no pain” to 10 = “worst pain” was used. The pain score at rest was evaluated three times per day (7 am, 3 pm and 11 pm), and data were collected and maintained in our hospital information system.

### Statistical analysis

Categorical variables were evaluated by the χ2 test. Continuous data were expressed as the mean ± standard deviation (SD). Before comparison, continuous variables of two groups were examined by the Levene test. When variances were not equal, the Brown-Forsythe test was performed, and when variances were equal, one way ANOVA was employed. Analyses were performed using the SPSS statistical package, version 20.0 (SPSS Inc., Chicago, IL, USA).

## Results

As shown in Tables 1, 270 male and 42 female patients aged from 40 to 76 years (64.42 ± 4.96 years) were included in this study. Based on the NCCN clinical practice guidelines [3], 203 patients underwent esophagogastric anastomosis through the right thoracic cavity, and 109 patients underwent surgery through the left thoracic cavity. According to whether an MDT was placed, enrolled patients were divided into two groups. A total of 138 patients with only one 26-Fr pleural drainage tube were divided into the “Control Group”, whereas 174 patients with a pleural drainage tube and an additional 26-Fr MDT were divided into the “Tube Group”. All patients received homogeneous postoperative care and nutrition support according to the NCCN clinical practice guidelines [3]. Among the recruited 312 patients, 26 patients (8.3%) suffered from AL, including 11 patients (8.0%) in the Control Group and 15 patients (8.6%) in the Tube Group. No significant differences were observed in the AL rate between the two groups (*P* = 0.837), which demonstrated that placing an MDT did not change the incidence of AL. Regarding postoperative pain associated with the drainage tube, patients in the Tube Group had a similar pain score on post-operative day 1 (*P* = 0.629), 2 (*P* = 0.347), 3 (*P* = 0.157), 4 (*P* = 0.799) and 5 (*P* = 0.190) when compared with patients in the Control Group.

**Table 1:** Demographic and perioperative features of the patients

	Control Group	Tube Group	***P*** value	Total
**Age**	64.51 ± 5.13	64.36 ± 4.83	0.790	64.42 ± 4.96
**Gender**			0.739	
Male	118 (85.5%)	152 (87.4%)		270 (86.5%)
Female	20 (14.5%)	22 (12.6%)		42 (13.5%)
**Smoking**			0.204	
No	25 (18.1%)	22 (12.6%)		47 (15.1%)
Yes	113 (81.9%)	152 (87.4)		256 (84.9%)
**Surgical approach**			0.812	
Right	91 (65.9%)	112 (64.4%)		203 (65.1%)
Left	47 (34.1%)	62 (35.6%)		109 (34.9%)
**Anastomic leakage**			0.837	
No	127 (92.0%)	159 (91.4%)		286 (91.7%)
Yes	11 (8.0%)	15 (8.6%)		26 (8.3%)
Postoperative pain score (NRS^*^)				
Day 1	3.94 ± 1.27	4.01 ± 1.25	0.629	3.98 ± 1.26
Day 2	3.30 ± 1.04	3.41 ± 0.90	0.347	3.36 ± 0.97
Day 3	2.86 ± 0.86	2.98 ± 0.69	0.157	2.93 ± 0.77
Day 4	2.78 ± 0.96	2.76 ± 0.63	0.799	2.77 ± 0.79
Day 5	2.16 ± 0.70	2.26 ± 0.64	0.190	2.21 ± 0.66

^*^ NRS: Numerical Rating Scale

When AL occurred, hyperpyrexia (T ≧ 38.5 °C) and abnormal drainage fluid from the MDT or pleural drainage tube, including a rapidly-increased drainage volume and a change in color and smell, were the earliest and most common symptoms. In general, the drainage volume would suddenly and greatly exceed that of the previous day, with an increment of more than 100 to 200 ml per day. Furthermore, the drainage fluid would become feculent and rancid. Table 2 shows that when AL occurred, hyperpyrexia was observed in all patients. In addition, abnormal drainage fluid was observed in all patients in the tube group, and only in 7 patients (7/11, 63.6%) in the control group.

**Table 2:** Treatments and outcomes of the patients with AL

	Control Group	Tube Group	*P* value
**Occurence time of hyperpyrexia (Day)**	5.27 ± 2.35	5.80 ± 1.87	0.693
**Abnormal drainage fluid**			
Number of patients	7 (63.6%)	15 (100%)	0.022
Occurence time (Day)	6.86 ± 1.35	3.94 ± 0.88	
AL^*^ **management**			
Conservative treatment	4 (36.4%)	12 (80.0%)	0.032
Re-operation	3 (27.3%)	0 (0%)	
Endoscopic treatment	4 (36.4%)	3 (20.0%)	
Outcome of AL^*^			
Patients recovered	8 (72.7%)	14 (93.3%)	
Hospital stay after AL^*^ (Recovery time)	86.50 ± 28.52	56.64 ± 23.64	0.015
Motality	3 (27.3%)	1 (6.7%)	0.188

^*^ AL: Anastomotic Leakage

In the tube group, the presentation of hyperpyrexia had a median of 5.80 ± 1.87 days after surgery, which was later than the abnormal drainage fluid from the MDT (3.94 ± 0.88 days). However, in the control group, abnormal drainage fluid from the pleural drainage tube had a median of 6.86 ± 1.35 days after surgery in the aforementioned 7 patients. This was intermittent and occurred later than hyperpyrexia, which appeared on a median of 5.27 ± 2.35 days after surgery. When persistent hyperpyrexia or abnormal drainage fluid was observed, AL was highly suspected and esophageal iodolography was performed to confirm the diagnosis (Fig. 1). Taken together, these results suggested that abnormal drainage fluid was more likely to appear in the MDT than in the pleural drainage tube (*P* = 0.022), and that occurrence of an abnormal drainage fluid in the MDT occurred earlier than hyperpyrexia. Thus, placement of an MDT could be a more sensitive and specific method for early diagnosis of AL.

Next, the therapeutic effect of the MDT was analyzed. In the tube group, the MDT was placed next to the anastomotic stoma during surgery, and was usually located in the abscess cavity. There was no need to place another drainage tube after AL occurred. After the diagnosis was confirmed, patients in the tube group received a normal saline flush twice a day through the MDT, enteral and parenteral nutrition support, and anti-infective therapy. Among them, 12 patients (80.0%) recovered without any other treatment, 3 patients (20.0%) received endoscopic therapy, including self-expandable metallic stents, and over-the-scope-clips. However, in the control group, the pleural drainage tube was not close to the anastomotic stoma. Therefore, abnormal drainage fluid was observed in only seven patients (63.6%). All patients in the control group underwent CT-guided or ultrasound-guided chest tube placement to manage the abscess cavity. Three patients (27.3%) still required re-operation to eliminate the abscess cavity, and another drainage tube was placed next to the anastomotic stoma, and a jejunostomy tube was placed for intra-intestinal nutrition, four patients (36.4%) received endoscopic therapy, and only four patients (36.4%) recovered with conservative therapy alone. Furthermore, the recovery time of patients in the tube group (56.64 ± 23.64) was significantly shorter compared to that of patients in the control group (86.50 ± 28.52 days) (*P* = 0.015). Thus, these results suggested that placement of an MDT could be convenient for drainage and cleaning of the abscess cavity, accelerated the recovery and reduced complications and the re-operation rate in patients suffering from AL.

Finally, the mortality between the two groups was analyzed. Among the 26 patients with AL, 1 patient in the tube group (6.7%) died of severe anastomotic hemorrhage, while 3 patients (27.3%) died of respiratory failure or systemic infection secondary to empyema in the control group, suggesting that placement of an MDT reduced the mortality rate after AL. No statistical differences were observed in mortality associated with AL between two groups (*P* = 0.188), perhaps because the number of AL cases in this study was limited.

## Discussion

The occurrence of AL after esophagectomy is a severe postoperative complication with a potential poor prognosis. In recent years, a wide range of measures to prevent AL has been suggested, including improvement in anastomotic techniques [16–18] and gastric tube formation [19, 20], utilization of a pedicled omental flap [21, 22] or mobilized pleura [23], thereby avoiding excessive tension at the anastomosis [24], intraoperative perfusion monitor [25, 26], efficient gastric decompression [27], and meticulous perioperative management. Nevertheless, only few interventions are supported by strong clinical evidence and AL is still been an important postoperative complication [9].

Because of the lack of typical symptoms and an effective real-time monitoring method, early diagnosis of introthoracic esophageal AL remains a problem. A wide variety of clinical manifestations has been included as early symptoms to diagnose AL, such as hyperpyrexia, thoracodynia, chest distress, and tachycardia [9–13]. According to the literature, hyperpyrexia, which results from a systemic infection secondary to the sepsis cavity around the anastomotic fistula, may be the earliest symptom of AL [9, 24, 28]. However, sometimes the first symptom is mild tachycardia or atrial fibrillation [10, 13]. Unfortunately, the occurrence of hyperpyrexia or tachycardia is nonspecific for diagnosis and prone to be neglected in clinical practice. These symptoms usually appear several hours or even several days later than AL. In contrast, abnormal liquid from the pleural drainage tube, including the rapidly increased drainage volume and the change in color and smell, can be a specific symptom for AL diagnosis. However, due to postoperative pleural adhesion and the distance between the anastomotic stoma and the pleural drainage tube, it also appears relatively late or, in most cases never occurs. To solve the problems mentioned above, we hypothesized that, once AL occurs, increased and feculent liquid could be immediately drained through a tube, which was placed during the operation next to the anastomic stoma in the esophagus bed, and could be easily observed for diagnosis. As a result, in this study, the occurrence of abnormal fluid from the MDT occurred earlier than hyperpyrexia. Therefore, placement of an MDT provides a new method for earlier and specific AL diagnosis.

As for AL treatment, there is currently no widely-accepted or standardized strategy. In general, the basic principles of treatments are the closure of the anastomotic fistula, containment of the leakage, drainage of fluid collections, and effective control of an infection [9]. Re-operation is one of the most effective treatment methods to manage an anastomotic fistula. However, because of the poor physical status of patients with AL, even in the absence of a consensus guideline, current strategies are still prone to shift from aggressive surgery to more conservative approaches [14], such as endoscopic treatment [15]. In previous studies, self-expandable metallic stents have been applied through endoscopy to cover the fistula and reduce the leakage [29]. An over-the-scope-clip system could restrain leakage by directly closing the fistula [30, 31]. Other reports focus on endoluminal suturing techniques [32] or the use of sealants [33, 34] for AL treatment. Nevertheless, without complete drainage and clearance of the sepsis cavity, the efficacy of the aforementioned strategies would be significantly restricted. To address this problem, endoluminal vacuum therapy was developed to remove secretions by continuous negative pressure suction [35–37]. However, in most cases, the local infection around the anastomotic fistula was already in a severe state when this therapy was applied, and vacuum through esophageal lumen could not completely eliminate the sepsis cavity outside. In this study, an MDT was placed during surgery next to the anastomotic stoma. Data showed that placement of the MDT prevented the retention of fluid from the fistula, decreased bacterial contamination, alleviated local infections, and was convenient for flushing to promote granulation tissue proliferation and closing of the fistula.

In addition, postoperative pain and safety are major concerns for both surgeons and patients. In this study, no significant difference was observed in the recorded pain scores or the incidence of AL between the two groups. Thus, these findings suggested that placement of an MDT is an adequate method for early diagnosis and effective treatment of intrathoracic AL.

## Conclusions

In conclusion, placing an MDT in the esophagus bed during surgery next to the anastomotic stoma is a safe and effective method to diagnose and treat introthoracic AL. The MDT showed excellent sensitivity and specificity in early diagnosis of the leakage and provided an approach for complete drainage and thorough cleaning. Taken together, the MDT is strongly recommended as a routine procedure during esophagectomy.

## Data Availability

The datasets used and/or analysed during the current study are available from the corresponding author on reasonable request.

## Abbreviations

AL: Anastomotic Leakage
MDT: Mediastinal Drainage Tube
NCCN: National Comprehensive Cancer Network

## References

[1] Smyth EC, Lagergren J, Fitzgerald RC, Lordick F, Shah MA, Lagergren P (2017). Oesophageal cancer. Nat Rev Dis Primers.

[2] Torre LA, Siegel RL, Ward EM, Jemal A (2016). Global cancer incidence and mortality rates and trends–an update. Cancer Epidemiol Biomark Prev.

[3] Ajani JA, D'Amico TA, Bentrem DJ, Chao J, Corvera C, Das P (2019). Esophageal and Esophagogastric junction cancers, version 2.2019, NCCN clinical practice guidelines in oncology. J Natl Compr Cancer Netw.

[4] van Workum F, van der Maas J, van den Wildenberg FJ, Polat F, Kouwenhoven EA, van Det MJ, Nieuwenhuijzen GA (2017). Improved functional results after minimally invasive Esophagectomy: Intrathoracic versus cervical anastomosis. Ann Thorac Surg.

[5] Seesing MFJ, Gisbertz SS, Goense L, van Hillegersberg R, Kroon HM, Lagarde SM (2017). A propensity score matched analysis of open versus minimally invasive transthoracic Esophagectomy in the Netherlands. Ann Surg.

[6] Schmidt HM, Gisbertz SS, Moons J, Rouvelas I, Kauppi J, Brown A (2017). Defining benchmarks for transthoracic Esophagectomy: A multicenter analysis of Total minimally invasive Esophagectomy in Low risk patients. Ann Surg.

[7] Low DE, Kuppusamy MK, Alderson D, Cecconello I, Chang AC, Darling G (2019). Benchmarking complications associated with Esophagectomy. Ann Surg.

[8] Kassis ES, Kosinski AS, Ross P, Koppes KE, Donahue JM, Daniel VC (2013). Predictors of anastomotic leak after Esophagectomy: an analysis of the Society of Thoracic Surgeons general thoracic database. Ann Thorac Surg.

[9] Fabbi M, Hagens ERC, van Berge Henegouwen MI, Gisbertz SS. Anastomotic leakage after esophagectomy for esophageal cancer: definitions, diagnostics, and treatment [published online ahead of print, 2020 Jun 1]. Dis Esophagus. 2020:doaa039. 10.1093/dote/doaa039. 10.1093/dote/doaa039 PMC7801633 32476017

[10] Stippel DL, Taylan C, Schröder W, Beckurts KT, Hölscher AH (2005). supraventricular tachyarrhythmia as early indicator of a complicated course after esophagectomy. Dis Esophagus.

[11] van Heijl M, van Wijngaarden AK, Lagarde SM, Busch OR, van Lanschot JJ, van Berge Henegouwen MI (2010). Intrathoracic manifestations of cervical anastomotic leaks after transhiatal and transthoracic oesophagectomy. Br J Surg.

[12] Korst RJ, Port JL, Lee PC, Altorki NK (2005). Intrathoracic manifestations of cervical anastomotic leaks after transthoracic esophagectomy for carcinoma. Ann Thorac Surg.

[13] Kechagias A, van Rossum PSN, Ruurda JP, van Hillegersberg R (2016). Ischemic conditioning of the stomach in the prevention of Esophagogastric anastomotic leakage after Esophagectomy. Ann Thorac Surg.

[14] Hagens ERC, Anderegg MCJ, van Berge Henegouwen MI, Gisbertz SS (2018). International survey on the Management of Anastomotic Leakage after Esophageal Resection. Ann Thorac Surg.

[15] Watkins JR, Farivar AS (2018). Endoluminal therapies for esophageal perforations and leaks. Thorac Surg Clin.

[16] Haverkamp L, Seesing MF, Ruurda JP, Boone JV, Hillegersberg R (2017). Worldwide trends in surgical techniques in the treatment of esophageal and gastroesophageal junction cancer. Dis Esophagus.

[17] Zhou D, Liu QX, Deng XF, Min JX, Dai JG. Comparison of two different mechanical esophagogastric anastomosis in esophageal cancer patients: a meta-analysis. J Cardiothorac Surg. 2015;10:67. Published 2015 May 8. doi:10.1186/s13019-015-0271-4. 10.1186/s13019-015-0271-4 PMC4456702 25952323

[18] Deng XF, Liu QX, Zhou D, Min JX, Dai JG (2015). Hand-sewn vs linearly stapled esophagogastric anastomosis for esophageal cancer: a meta-analysis. World J Gastroenterol.

[19] Zhang W, Yu D, Peng J, Xu J, Wei Y. Gastric-tube versus whole-stomach esophagectomy for esophageal cancer: A systematic review and meta-analysis. PLoS One. 2017;12(3):e0173416. Published 2017 Mar 7. doi:10.1371/journal.pone.0173416. 10.1371/journal.pone.0173416 PMC5340360 28267808

[20] Laméris W, Eshuis WJ, Cuesta MA, Gisbertz SS, van Berge Henegouwen MI (2019). Optimal mobilization of the stomach and the best place in the gastric tube for intrathoracic anastomosis. J Thorac Dis.

[21] Zhang QX, Magovern CJ, Mack CA, Budenbender KT, Ko W, Rosengart TK (1997). Vascular endothelial growth factor is the major angiogenic factor in omentum: mechanism of the omentum-mediated angiogenesis. J Surg Res.

[22] Yuan Y, Zeng X, Hu Y, Xie T, Zhao Y. Omentoplasty for oesophagogastrostomy after oesophagectomy. Cochrane Database Syst Rev. 2014;(10):CD008446. Published 2014 Oct 2. doi:10.1002/14651858.CD008446.pub3. 10.1002/14651858.CD008446.pub3 PMC10961160 25274134

[23] Asteriou C, Barbetakis N, Lalountas M, Kleontas A, Tsilikas C (2011). Modified pleural tenting for prevention of anastomotic leak after Ivor Lewis esophagogastrectomy. Ann Surg Oncol.

[24] Vetter D, Gutschow CA. Strategies to prevent anastomotic leakage after esophagectomy and gastric conduit reconstruction [published online ahead of print, 2020 Jul 10]. Langenbecks Arch Surg. 2020. 10.1007/s00423-020-01926-8, 10.1007/s00423-020-01926-8. 10.1007/s00423-020-01926-8 PMC7686179 32651652

[25] Köhler H, Jansen-Winkeln B, Chalopin C, Gockel I (2019). Hyperspectral imaging as a new optical method for the measurement of gastric conduit perfusion. Dis Esophagus.

[26] Slooter MD, Eshuis WJ, Cuesta MA, Gisbertz SS, van Berge Henegouwen MI (2019). Fluorescent imaging using indocyanine green during esophagectomy to prevent surgical morbidity: a systematic review and meta-analysis. J Thorac Dis.

[27] Weijs TJ, Kumagai K, Berkelmans GH, Nieuwenhuijzen GA, Nilsson M, Luyer MD (2017). Nasogastric decompression following esophagectomy: a systematic literature review and meta-analysis. Dis Esophagus.

[28] Tang H, Xue L, Hong J, Tao X, Xu Z, Wu B (2012). A method for early diagnosis and treatment of intrathoracic esophageal anastomotic leakage: prophylactic placement of a drainage tube adjacent to the anastomosis. J Gastrointest Surg.

[29] Dasari BV, Neely D, Kennedy A (2014). The role of esophageal stents in the management of esophageal anastomotic leaks and benign esophageal perforations. Ann Surg.

[30] Hagel AF, Naegel A, Lindner AS, Kessler H, Matzel K, Dauth W, Neurath MF, Raithel M (2012). Over-the-scope clip application yields a high rate of closure in gastrointestinal perforations and may reduce emergency surgery. J Gastrointest Surg.

[31] Haito-Chavez Y, Law JK, Kratt T, Arezzo A, Verra M, Morino M, Sharaiha RZ, Poley JW, Kahaleh M, Thompson CC, Ryan MB, Choksi N, Elmunzer BJ, Gosain S, Goldberg EM, Modayil RJ, Stavropoulos SN, Schembre DB, DiMaio CJ, Chandrasekhara V, Hasan MK, Varadarajulu S, Hawes R, Gomez V, Woodward TA, Rubel-Cohen S, Fluxa F, Vleggaar FP, Akshintala VS, Raju GS, Khashab MA (2014). International multicenter experience with an over-the-scope clipping device for endoscopic management of GI defects (with video). Gastrointest Endosc.

[32] Gaur P, Lyons C, Malik TM, Kim MP, Blackmon SH (2015). Endoluminal suturing of an anastomotic leak. Ann Thorac Surg.

[33] Ojima T, Nakamura M, Nakamori M, Katsuda M, Hayata K, Tsuji T (2018). Endoscopic treatment of esophageal fistulas after esophagectomy with injection of an alpha-cyanoacrylate monomer: a phase II study. Endosc Int Open.

[34] Ojima T, Nakamori M, Nakamura M, Katsuda M, Iida T, Hayata K, et, al. Successful treatment of esophageal fistulas with endoscopic injection of alpha-cyanoacrylate monomer. Endoscopy. 2014;46 Suppl 1 UCTN:E62-E63. doi:10.1055/s-0033-1359159. 10.1055/s-0033-1359159 24523186

[35] Mennigen R, Senninger N, Laukoetter MG (2014). Novel treatment options for perforations of the upper gastrointestinal tract: endoscopic vacuum therapy and over-the-scope clips. World J Gastroenterol.

[36] Laukoetter MG, Mennigen R, Neumann PA, Dhayat S, Horst G, Palmes D (2017). Et, al. Successful closure of defects in the upper gastrointestinal tract by endoscopic vacuum therapy (EVT): a prospective cohort study. Surg Endosc.

[37] Newton NJ, Sharrock A, Rickard R, Mughal M (2017). Systematic review of the use of endo-luminal topical negative pressure in oesophageal leaks and perforations. Dis Esophagus.

